# A rare case of nasal septal schwannoma: Case report and literature review

**DOI:** 10.1016/j.ijscr.2019.01.027

**Published:** 2019-01-30

**Authors:** Wejdan Alrasheed, Ali Almomen, Abdulrahman Alkhatib

**Affiliations:** a Imam Abdulrahman Bin Faisal University, Dammam, Saudi Arabia; b Department of ENT, King Fahad Specialist Hospital, Dammam, Saudi Arabia

**Keywords:** Nasal mass, Nasal septum, Schwannoma

## Abstract

Introduction: Sinonasal schwannoma accounts for only 4% of head and neck nerve sheath tumors. Schwannoma of the nasal septum is still the rarest, with only few cases previously reported.

Case presentation: This study presents the case of a 64-year-old Saudi female of the Eastern Province who presented a several-month history of unilateral progressive nasal obstruction and recurrent episodes of epistaxis. The diagnosis of nasal septal schwannoma was made on the basis of a histopathological examination. The case was managed successfully by endoscopic endonasal excision with no postoperative recurrence.

Discussion: Nasal septal schwannoma is rare and has only 32 reported cases. Diagnosis primarily depends on histopathological examination. Endoscopic endonasal excision of the mass is considered the ideal treatment for such pathology.

Conclusion: Schwannoma of the nasal septum is rare. However, it should be included as a differential diagnosis in a patient presenting with a unilateral nasal mass.

## Highlights

• Schwannoma is a benign nerve sheath tumor that can arise from any myelinated nerve fiber.
• Nasal septal schwannoma is rare, and only 32 cases have been reported in the literature.
• The diagnosis of the nasal septal schwannoma primarily depends on histopathological examination.
• Endoscopic endonasal excision of the mass is considered the ideal treatment for such pathology, with rare postoperative recurrence.

## 1 Introduction

Schwannoma is a benign nerve sheath tumor that can arise from any myelinated nerve fiber. The most common site of schwannoma in the head and neck region is the eighth cranial nerve (vestibulocochlear nerve). Other sites include the scalp, face, parotid gland, oral cavity, pharynx, larynx, and trachea [1]. Sinonasal schwannoma accounts for only 4% of head and neck nerve sheath tumors. Schwannoma of the nasal septum is still the rarest, with only few cases previously reported [2]. The diagnosis of nasal septal schwannoma is based on histopathological findings due to a lack of characteristic radiological features [3]. This case study has been reported according to Surgical Case Reports criteria [4].

## 2 Case presentation

A 64-year-old Saudi female of the Eastern Province was referred to our institute with a several-month history of unilateral progressive nasal obstruction and recurrent episodes of epistaxis. There was no history of anosmia, nasal discharge, nasal congestion, sneezing, facial pain, or headache. Her past medical history included a known case of hypertension and diabetes. Her family history was otherwise unremarkable.

**Fig. 1:**
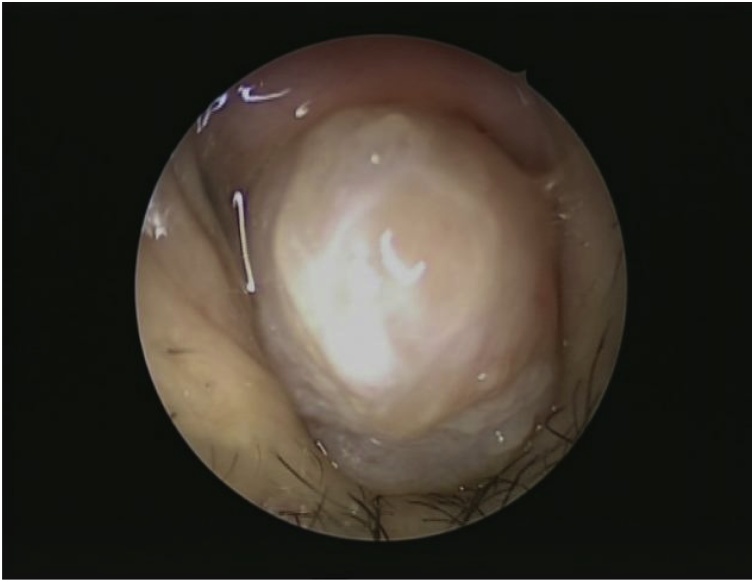
Endoscopic examination of the nose showed a large, left-sided nasal polypoidal mass occluding the entire left nasal cavity with smooth overlying mucosa.

A rigid endoscopic examination of the nose showed a large, left-sided nasal polypoidal mass with smooth overlying mucosa that occluded the whole left nasal cavity (Fig. 1). The remaining ear, nose, throat, head, and neck examinations were normal, and there was no palpable lymphadenopathy.

**Fig. 2:**
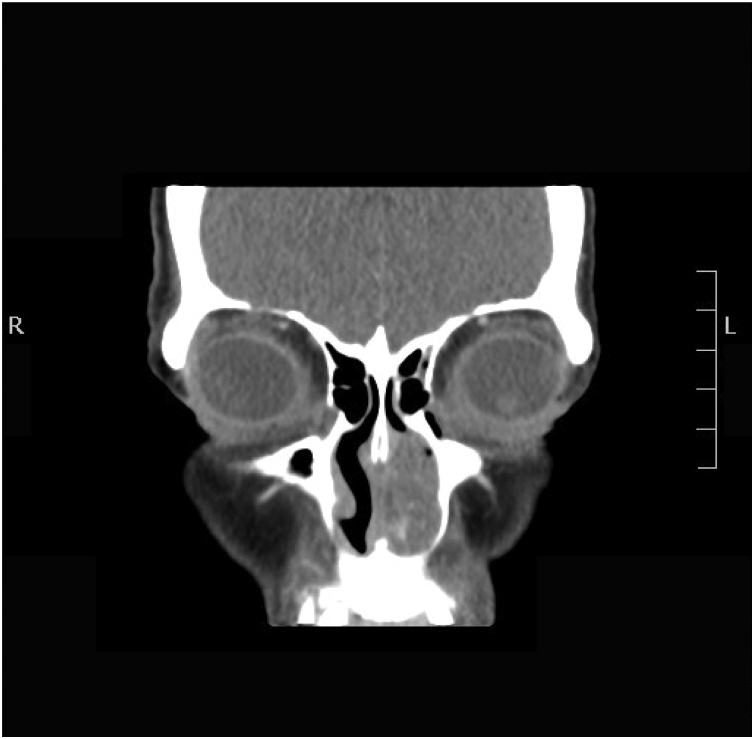
CT scan of the paranasal sinuses with contrast. The coronal section showed a left anterior lobulated nasal mass of undetermined origin at the level of the cartilaginous part of the nasal septum with mild heterogeneous post IV contrast enhancement.

A CT scan of the paranasal sinuses with contrast showed a left anterior lobulated nasal mass of undetermined origin at the level of the cartilaginous part of the nasal septum with mild heterogeneous post IV contrast enhancement and bone remodeling. There was no extension to the paranasal sinuses, and no obvious bone invasion was observed (Fig. 2).

**Fig. 3:**
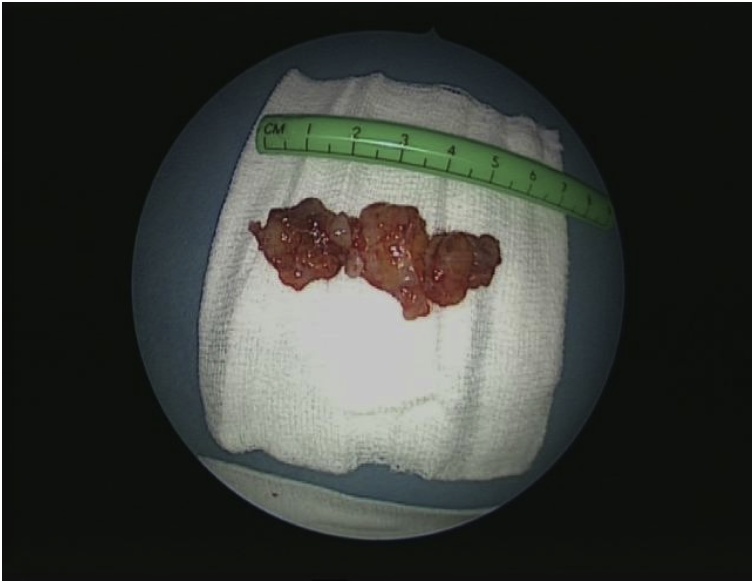
Gross image of the mass revealed multiple fragments of soft to firm tan-gray tissue.

Endonasal endoscopic excision of the left nasal cavity mass was performed under general anesthesia. Intraoperative findings demonstrated a semifirm vascular mass attached to the anterior face of the middle turbinate and septum at the same level. The mass was excised completely from its attachment, and the pedicle was cauterized. Histological examination of the mass revealed three fragments of soft to firm tan-gray tissue, the largest measuring 1.8 × 1.6 × 0.6 cm, and the smallest measuring 0.8 × 0.6 × 0.5 cm (Fig. 3). The lesion was positive for vimentin and S100 and negative for desmin, NSE, and SMA. The final histopathology confirmed the diagnosis of schwannoma of the nasal septum.

The patient has received regular follow-up care for three years with no recurrence.

## 3 Discussion

Nasal septal schwannoma is rare, and only 32 cases have been reported in the literature [5]. Schwannoma is predominantly observed in the forty to sixty year adult age group with no sex or racial predilection [6]. Nasal septal schwannoma typically involves the posterior part of the septum and is likely to arise from the nasopalatine branch of the trigeminal nerve. It usually presents with nasal obstruction, anosmia, deformity of the nasal pyramid, headache, and epistaxis [7].

The differential diagnosis for unilateral nasal mass includes nasal polyposis (22%), antrochoanal polyps (19%), chronic rhinosinusitis (13%), concha bullosa (11%), inverted papilloma (6%), and retention cysts (6%). Other less common potential causes include fibrous dysplasia, mucocele, lymphoma, schwannoma, ameloblastoma, pleomorphic adenoma, myxoma, and squamous cell carcinoma [2]. Due to the wide variety of potential pathologies in unilateral nasal obstruction, it is difficult to make a diagnosis on the basis of imaging alone [8].

CT scan findings are not specific for a schwannoma diagnosis. However, CT is helpful in evaluating the tumor’s origin and extent. MRI is favored over CT in differentiating tumors from inflammatory disorders and normal tissue; additionally, MRI provides information regarding intracranial invasion of the tumor [9]. MRI has also shown specific characteristic findings of a nerve sheath tumor in previous cases, which include the target sign and fascicular sign on the T2-weighted sequence [8,10,11].

**Fig. 4:**
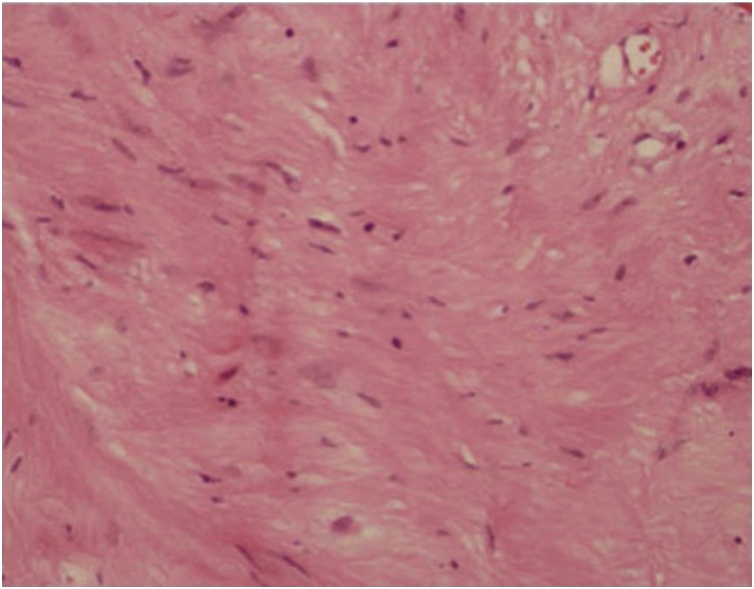
Histopathological examination showed high cellular density and a palisading pattern of the tumor cells (*40 power, H&E stained microscopic slide picture).

**Fig. 5:**
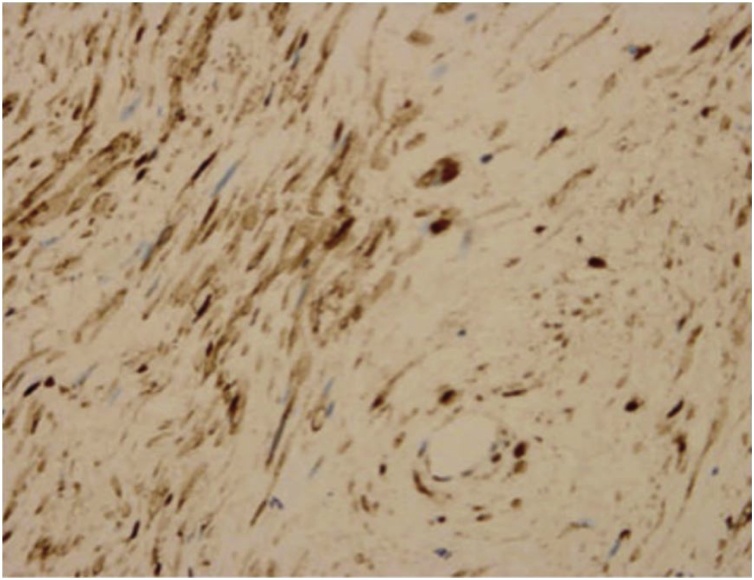
Immunohistochemistry showed tumor cells that are strongly positive for S100.

Because imaging is non-specific for schwannoma, nasal septal schwannoma diagnosis primarily depends on biopsy or complete excision of the mass. Macroscopically, schwannomas appear as well circumscribed, encapsulated, cystic masses that are connected to the nerve tissue. Microscopically, schwannomas are classified into two types: Antoni A and Antoni B patterns. Antoni A patterns are composed of spindle cells organized as cellular areas with nuclear palisading (Fig. 4). Antoni B patterns are characterized by disorganized, loose myxoid stroma with few spindle cells [[12], [13], [14]]. If a schwannoma does not demonstrate characteristic histopathological features, immunohistochemical staining for S100 proteins may aid in schwannoma diagnosis (Fig. 5). Additionally, calretinin is a useful marker in differentiating schwannoma from neurofibroma [13].

The preferred treatment for nasal septal schwannoma is surgical resection of the mass, which may be achieved by lateral rhinotomy or endoscopic endonasal surgery [7]. The endoscopic endonasal approach with or without image guidance is the standard surgical approach for tumor removal. Advantages include avoidance of external incision, excellent visualization, minimal invasivity, a shorter hospital stay, and lower morbidities compared to external surgical approaches. The condition is typically curative with rare postoperative recurrence [15].

## 4 Conclusion

Schwannoma arising from the nasal septum is rare. However, it should be included as a differential diagnosis in a patient presenting with a unilateral nasal mass. Histopathological examination is the gold standard test for diagnosis. Furthermore, endoscopic endonasal excision of the mass is the ideal treatment because it is a minimally invasive approach with improved visualization, lower morbidities, shorter hospital stays and external scar avoidance.

## Conflicts of interest

There are no conflicts of interest.

## Funding

None.

## Ethical approval

Ethical approval is exempted for the case report at our institution.

## Consent

Written informed consent was obtained from the patient for publication of this case report and accompanying images. A copy of the written consent is available for review by the Editor-in-Chief of this journal on request.

## Author contribution

Wejdan Alrasheed: manuscript draft and final edits.

Ali Almomen: operating surgeon, data analysis and interpretation and critical revision of the manuscript.

Abdulrahman Alkhatib: data collection and literature review.

## Registration of research studies

Not applicable.

## Guarantor

The corresponding author.

## Provenance and peer review

Not commissioned, externally peer-reviewed.
